# Enhancing oil production and harvest by combining the marine alga *Nannochloropsis oceanica* and the oleaginous fungus *Mortierella elongata*

**DOI:** 10.1186/s13068-018-1172-2

**Published:** 2018-06-22

**Authors:** Zhi-Yan Du, Jonathan Alvaro, Brennan Hyden, Krzysztof Zienkiewicz, Nils Benning, Agnieszka Zienkiewicz, Gregory Bonito, Christoph Benning

**Affiliations:** 1 Department of Energy-Plant Research Laboratory, Michigan State University, East Lansing, MI 48824 USA; 2 Department of Biochemistry and Molecular Biology, Michigan State University, East Lansing, MI 48824 USA; 3 Department of Plant Biochemistry, Albrecht-von-Haller-Institute for Plant Sciences, Georg-August-University, 37073 Goettingen, Germany; 4 Department of Plant, Soil and Microbial Sciences, Michigan State University, East Lansing, MI 48824 USA; 5 Great Lakes Bioenergy Research Center, Michigan State University, East Lansing, MI 48824 USA; 6 Department of Plant Biology, Michigan State University, East Lansing, MI 48824 USA

**Keywords:** *Nannochloropsis*, *Mortierella*, Bio-flocculation, Polyunsaturated fatty acid, Triacylglycerol, Photobioreactor, Microalgae, Filamentous fungi, Cell–wall interaction, Biofuel, Nitrogen starvation

## Abstract

**Background:**

Although microalgal biofuels have potential advantages over conventional fossil fuels, high production costs limit their application in the market. We developed bio-flocculation and incubation methods for the marine alga, *Nannochloropsis oceanica* CCMP1779, and the oleaginous fungus, *Mortierella elongata* AG77, resulting in increased oil productivity.

**Results:**

By growing separately and then combining the cells, the *M. elongata* mycelium could efficiently capture *N. oceanica* due to an intricate cellular interaction between the two species leading to bio-flocculation. Use of a high-salt culture medium induced accumulation of triacylglycerol (TAG) and enhanced the contents of polyunsaturated fatty acids (PUFAs) including arachidonic acid and docosahexaenoic acid in *M. elongata*. To increase TAG productivity in the alga, we developed an effective, reduced nitrogen-supply regime based on ammonium in environmental photobioreactors. Under optimized conditions, *N. oceanica* produced high levels of TAG that could be indirectly monitored by following chlorophyll content. Combining *N. oceanica* and *M. elongata* to initiate bio-flocculation yielded high levels of TAG and total fatty acids, with ~ 15 and 22% of total dry weight (DW), respectively, as well as high levels of PUFAs. Genetic engineering of *N. oceanica* for higher TAG content in nutrient-replete medium was accomplished by overexpressing *DGTT5*, a gene encoding the type II acyl-CoA:diacylglycerol acyltransferase 5. Combined with bio-flocculation, this approach led to increased production of TAG under nutrient-replete conditions (~ 10% of DW) compared to the wild type (~ 6% of DW).

**Conclusions:**

The combined use of *M. elongata* and *N. oceanica* with available genomes and genetic engineering tools for both species opens up new avenues to improve biofuel productivity and allows for the engineering of polyunsaturated fatty acids.

**Electronic supplementary material:**

The online version of this article (10.1186/s13068-018-1172-2) contains supplementary material, which is available to authorized users.

## Background

Plant and algal oils are among the most energy-dense naturally occurring compounds that can be used as feedstocks for biofuel products. Microalgae have been considered as promising sustainable feedstock for supplanting fossil fuels since the 1970s. Advantages of microalgae over other biofuel feedstocks include high oil yield, short generation times, low agricultural land requirements, reduced fresh water needs, and reduced greenhouse gas emissions during algal cultivation [1–3]. To improve lipid productivity of microalgae, many studies have been conducted in model microalgae such as *Chlamydomonas reinhardtii* [4, 5], and biotechnologically relevant species such as *Nannochloropsis* [6–9]. *Nannochloropsis* species have become a focus for lipid and biofuel research, because these marine microalgae grow fast in open ponds or photobioreactors, and can be grown in seawater with high yields of lipid—up to 60% of dry weight (DW) [10–12]. In addition, *Nannochloropsis* is enriched in high-value polyunsaturated fatty acids (PUFAs) such as omega-3 eicosapentenoic acid (EPA), and it has a small and compact haploid genome (~ 30 Mbp). In recent years, the genomes of many species and strains of *Nannochloropsis* including *N. gaditana* and *N. oceanica* have been sequenced [13–15], and genetic engineering methods have been developed for gene disruption, i.e. CRISPR–Cas9 [8, 16, 17], and to generate overproduction systems for triacylglycerols (TAGs) and other target molecules [18, 19].

In spite of these apparent advantages, the high cost of microalgal-based fuel production prevents its application in the market [20–22]. The major barriers for the cost-effective production of microalgal biofuels include (1) high cost for harvesting microalgae; (2) low oil content and suboptimal composition; (3) high cost of lipid extraction; and (4) impasses in sustainable nutrient supply. Among these barriers, harvesting microalgae is particularly challenging because of the small cell size (typically 2–20 μm) and low density (0.3–5 g L^−1^) of microalgae, which can account for up to 50% of the total cost of biofuel products [23–25]. Traditional harvesting methods include chemical flocculation using multivalent cations such as metal salts and cationic polymers to neutralize the negative charge on the surface of microalgal cell walls, filtration for relatively large algae (> 70 μm), sedimentation/floatation for species that either fall out of suspension or float without sufficient mixing, thermal drying, and centrifugation, which have high cost and energy consumption [24–26].

Recently, bio-flocculation of microalgae with living materials such as bacteria and fungi has gained interest because of their high efficiency and relatively low energy requirement [23, 27–29]. In addition, many bio-flocculants can be cultured with nutrients from industrial wastes [29, 30]. The gram-positive bacterium *Solibacillus silvestris* can efficiently flocculate *N. oceanica* DUT01 without the addition of chemical flocculants or high-energy inputs. The bacterial flocculant exhibits no growth effect on the algal cells, and it can be reused for lower cost in harvesting [31]. In addition, some bio-flocculants such as filamentous fungi *Aspergillus fumigatus* and *Mucor circinelloides* can accumulate 10–15% lipid per DW which can increase the total lipid yield [30]. However, these filamentous fungi are also human pathogens, making them unsuitable for use as bio-flocculants.

Here we screened oleaginous fungi with regard to their ability to flocculate *N. oceanica* CCMP1779, a marine alga with a sequenced genome, rapidly growing molecular engineering tool kit, and the ability to produce high levels of TAG [9, 13, 18]. We discovered that *N. oceanica* could be efficiently flocculated by *M. elongata* AG77, a soil fungus that accumulates high levels of TAG and arachidonic acid (ARA), and for which a sequenced genome is available [32]. We provide detailed insights into the physical interaction between *N. oceanica* and *M. elongata* AG77 and test approaches for increasing the TAG content in *N. oceanica* by optimizing growth conditions and genetic engineering approaches in combination with bio-flocculation to harvest algal cells.

## Results

### *N. oceanica* cells are captured by the *M. elongata* mycelium

**Fig. 1:**
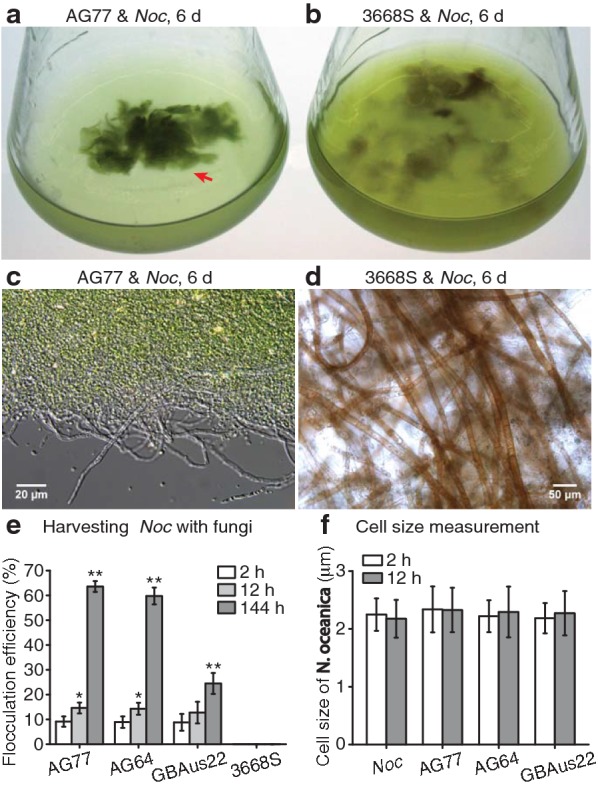
Harvesting *Nannochloropsis oceanica* by bio-flocculation with *Mortierella* fungi. **a**, **b** Co-culture of *N. oceanica* (*Noc*) with *M. elongata* AG77 (**a**), or *Morchella americana* 3668S (**b**). Fungal mycelium collected from PDB medium was added to a log-phase *Noc* culture grown in flasks and both were incubated for 6 days in flasks containing *f*/2 medium. The red arrow indicates green aggregates formed by AG77 mycelium and attached *Noc* cells. **c** *Noc* cells attached to AG77 mycelium as shown by differential interference contrast (DIC) microscopy. **d** No obvious attachment of *Noc* cells on the 3668S mycelium. **e** Bio-flocculation efficiency for harvesting Noc cells by cocultivation with *Mortierella elongata* AG77, *M. elongata* NVP64, and *M. gamsii* GBAus22 in *f*/2 medium. The bio-flocculation efficiency was determined by the cell density of uncaptured cells compared to that of a no-fungus *Noc* culture control. *Morchella* 3668S culture was used as a negative control. The results are the average of five biological replicates and error bars indicate standard deviation. Asterisks indicate significant differences relative to the 2-h co-cultures by paired-sample Student’s t-test (*P ≤ 0.05; **P ≤ 0.01). **f** Measurement of *Noc* cell size (diameter) in the *Noc* culture and alga-fungus co-culture

Fungi were incubated in potato dextrose broth (PDB). Fungal mycelium (~ 3 times of algal biomass) was added to the *N. oceanica* culture containing log-phase cells in *f*/2 medium in shaker flasks. After 6-day co-cultivation with *M. elongata, N. oceanica* cells aggregated in dense green clumps along the mycelium of the fungus (Fig. 1a). The interaction of *N. oceanica* with filamentous fungi appeared specific to *M. elongata,* as it was not observed in co-culture with *Morchella americana* 3668S (Fig. 1). Differential interference contrast (DIC) light microscopy showed dense numbers of *N. oceanica* cells attached to the *M. elongata* mycelium (Fig. 1c); in comparison, mycelium of *M. americana* hardly captured any algal cells (Fig. 1d). Three *Mortierella* strains, *M. elongata* AG77, *M. elongata* NVP64, and *M. gamsii* GBAus22 were used to test the flocculation efficiency for harvesting of *N. oceanica* with *M. americana* as a negative control. All three *Mortierella* isolates aggregated ~ 10% of algal cells after 2-h of co-culture and up to ~ 15% after 12 h (Fig. 1e). After 6-day co-cultivation, *M. elongata* AG77 and NVP64 captured ~ 60% of algal cells *M. gamsii* GBAus 22 captured ~ 25%. The short period of co-cultivation with fungi did not appear to affect the morphology of the algal cells and did not significantly change their diameter (Fig. 1f).

### Physical interaction between the cell walls of *N. oceanica* and *Mortierella* fungi

**Fig. 2:**
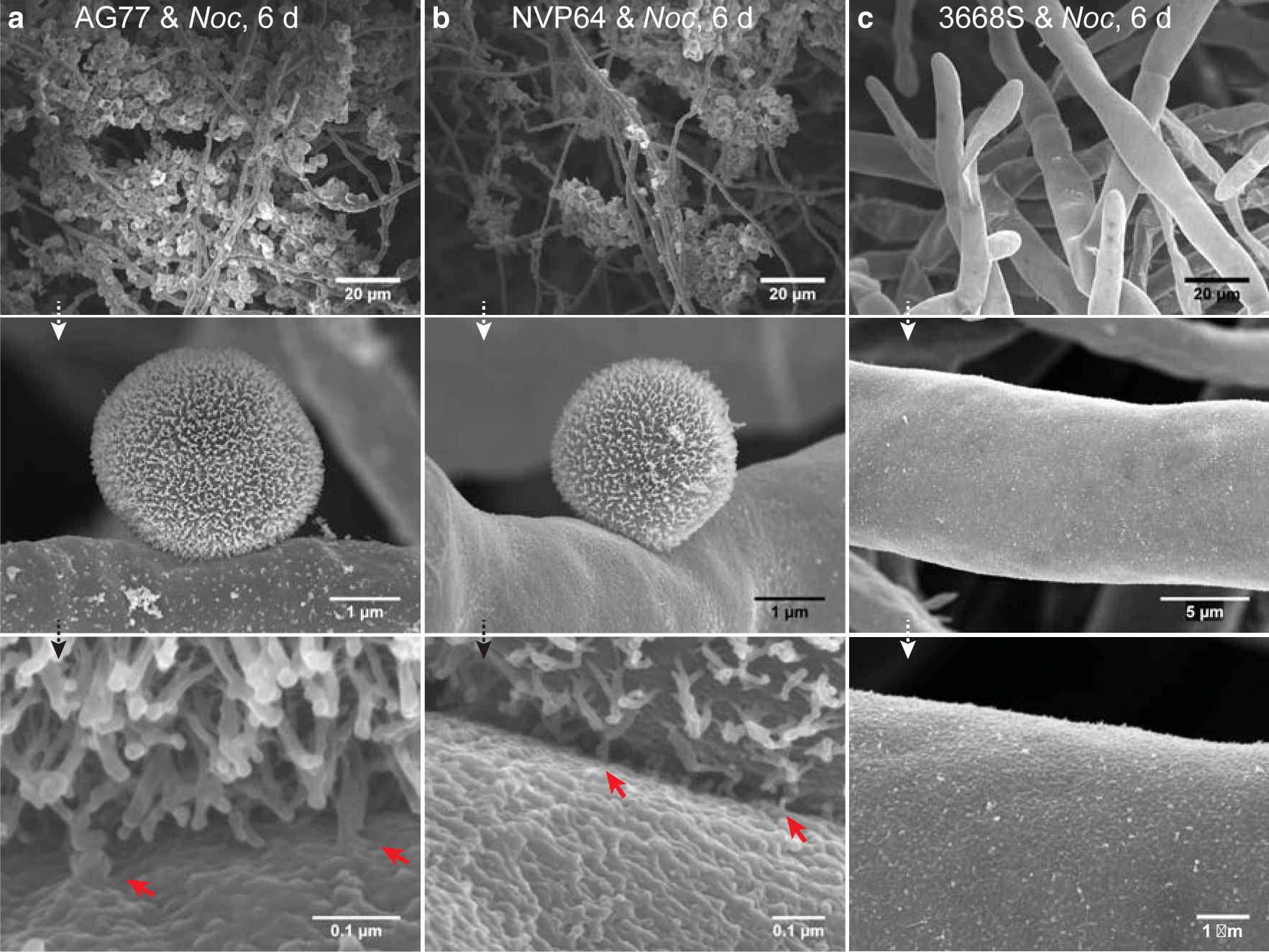
Interaction between *N. oceanica* and *Mortierella* mycelium co-cultivated in *f*/2 medium. Fungal mycelium was added to a log-phase *Noc* culture grown in a flask and then the mixture was incubated for 6 days in *f*/2 medium. Scanning electron microscopy was performed to investigate the interaction between *N. oceanica* (*Noc*) cells and *M. elongata* AG77 (**a**) and NVP64 (**b**). *Noc* cells are attached to the fungal mycelium as shown in the top panel. Higher magnification micrographs show that *Noc* cells have a highly structured cell wall with protrusions, with which they attach to the rough surface of the fungal cell wall. The red arrowheads indicate tube-like structures that connect the algal and fungal cell walls. **c** *Morchella americana* 3668S mycelium collected from a *Noc*-3668S culture after 6-day co-cultivation

Scanning electron microscopy (SEM) was performed to investigate the physical interaction between *N. oceanica* and *M. elongata* strains AG77 (Fig. 2a) and NVP64 (Fig. 2b). Low-magnification images (Fig. 2, top panels) showed an aggregation of algal cells around the fungal mycelium as seen in the light micrographs (Fig. 1c). Higher-magnification images displayed details of the physical interaction between the alga and fungus (Fig. 2, middle and bottom panels). Similar to the cell–wall structure of *N. gaditana* [33], *N. oceanica* has extensions on the outer layer of the cell wall, which are attached to the rugged surface of the fungal hyphae; irregular tube-like structures are formed between the algal and fungal cell walls, which very likely contribute to anchoring the algal cells to the mycelium. The *M. americana* strain 3668S, which has much thicker hyphae (10–20 μm in diameter) than the *M. elongata* strains AG77 and NVP64 (< 2 μm), showed no obvious capture of *N. oceanica* cells (Fig. 2c) or flocculation.

### Flocculation of *N. oceanica* with *Mortierella* fungi increases the yield of TAG and PUFAs

**Fig. 3:**
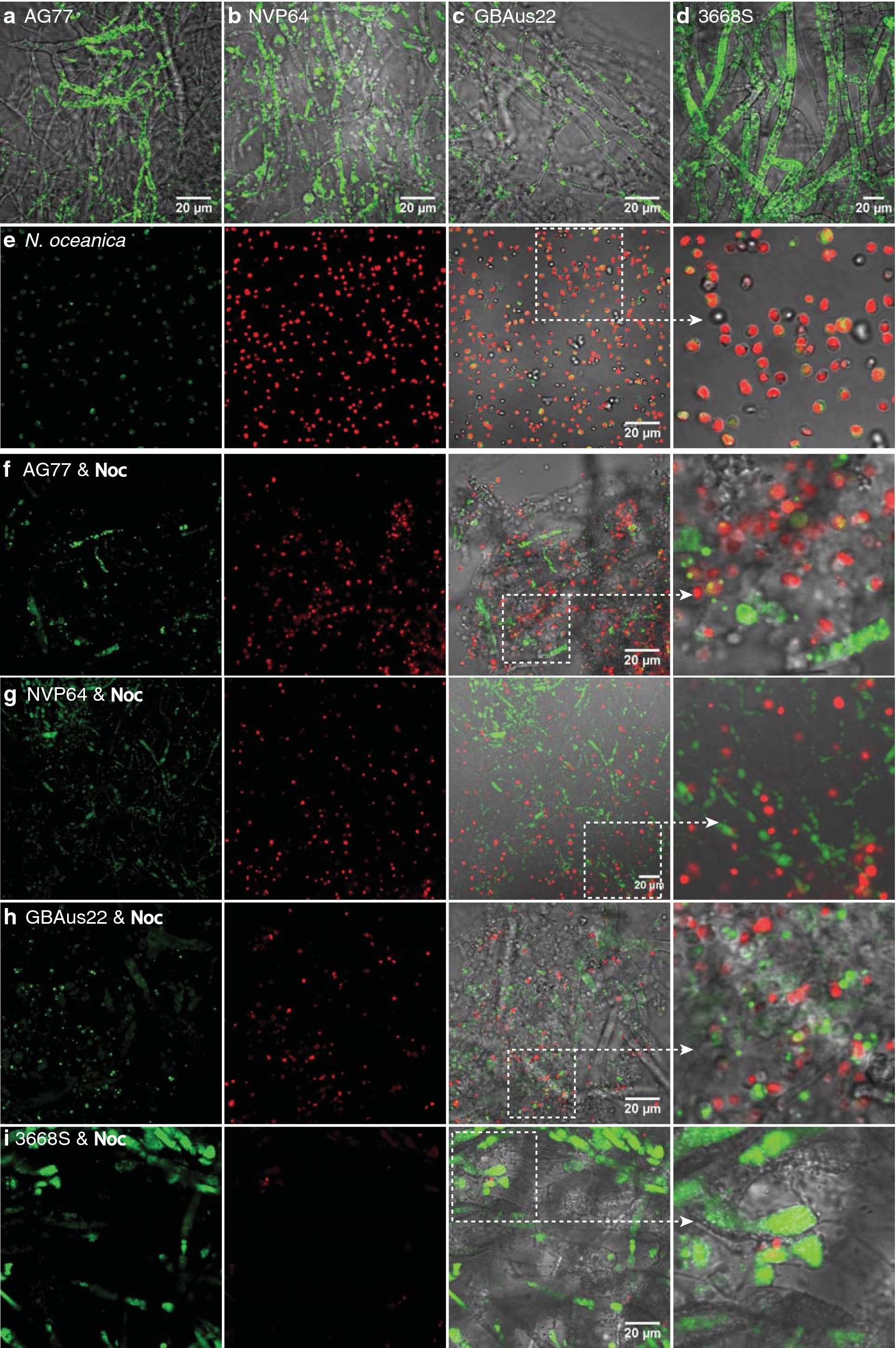
*Mortierella* fungi have more oil droplets than *N. oceanica* in *f*/2 medium. **a**–**d** Confocal micrographs showing lipid droplets in the fungal mycelium grown in flasks containing PDB medium. Green fluorescence indicates lipid droplets stained with BODIPY. **e** Lipid droplets in log-phase *N. oceanica* (*Noc*) cells grown in flasks containing *f*/2 medium. Red, autofluorescence of *Noc* chloroplast. **f**–**i** Lipid droplets in the algal and fungal cells after 6-day co-cultivation in *f*/2 medium. Fungal mycelium was added to the log-phase *Noc* culture for 6-day co-culture in flasks. AG77, *M. elongata* AG77; NVP64, *M. elongata* NVP64; GBAus22, *M. gamsii* GBAus22; 3668S, *Morchella americana* 3668S

**Fig. 4:**
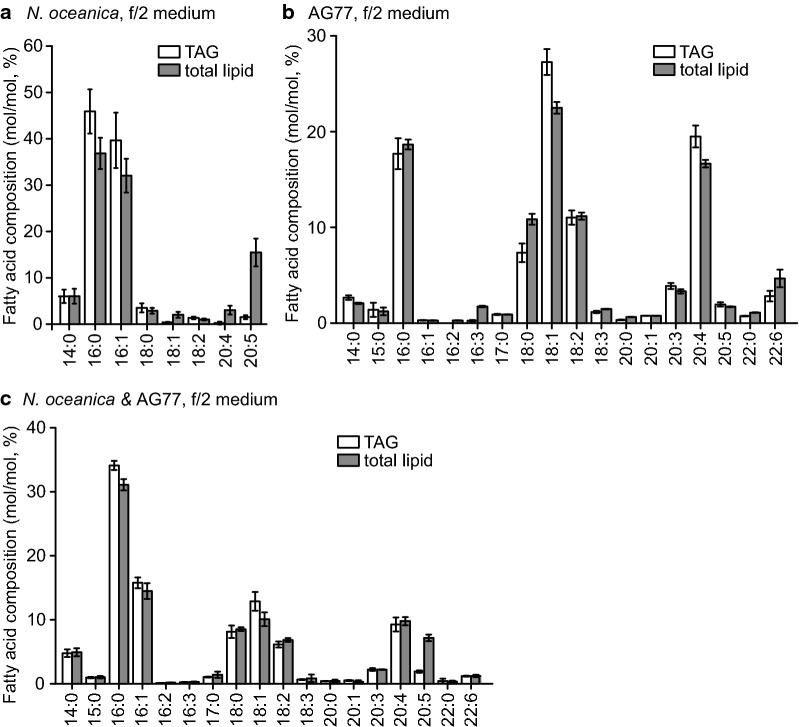
Fatty acid profiling of triacylglycerol (TAG) and total lipid in *Mortierella* fungi, *N. oceanica*, and algae–fungi aggregates after 6-day co-cultivation. **a** Fatty acid assays of triacylglycerol and total lipid in log-phase *N. oceanica* grown in shaker flask containing *f*/2 medium. Fatty acids are indicated with number of carbons: number of double bonds. Results are the average of five biological replicates with error bars indicating standard deviations (*n* = 5). **b** Fatty acid assays of *M. elongata* AG77 incubated in *f*/2 medium. Fungal mycelium was collected from PDB medium and then washed and added to *f*/2 medium and incubated for 6 days. *n* = 5. **c** Fatty acids of the algae–fungi aggregates after 6-day co-cultivation in flasks containing *f*/2 medium. *n* = 5

*Mortierella* fungi are known to produce high amounts of TAG and PUFAs including ARA [34, 35]. Indeed, numerous lipid droplets were observed in both *Mortierella* and *Morchella* fungi tested for algae flocculation (Fig. 3a–d). In contrast, *N. oceanica* had fewer and smaller lipid droplets when grown in nutrient-sufficient *f*/2 medium with or without fungi (Fig. 3e–i) in shaker flasks. Lipids were extracted and separated by thin-layer chromatography (TLC), and fatty acid methyl esters were quantified by gas chromatography and flame ionization detection (GC–FID) to determine the lipid and fatty acid composition. As shown in Table 1, *M. elongata* AG77 and *M. gamsii* GBAus22 had much higher contents of TAG, ARA, total PUFAs and total fatty acids, but less content of EPA compared to *N. oceanica*, which affects the final yield of these compounds in the alga-fungus aggregate. *N. oceanica* TAG is mainly composed of saturated and monounsaturated fatty acids such as C16:0 and C16:1 (Fig. 4a), whereas *Mortierella* fungi have more PUFAs, especially ARA (Fig. 4b). *N. oceanica* has more EPA in total lipid than in TAG (Fig. 4a), and the alga-fungus aggregate contains ~ 10% ARA and ~ 7% EPA of total lipid (Fig. 4c).

**Table 1:** Lipid contents of different strains grown in *f*/2 medium in shaker flasks (mg g^−1^ total dry weight)

Strains	Total fatty acid	TAG	ARA	EPA	Total PUFAs
*N. oceanica*	118.7 ± 18.4	15.1 ± 2.3	3.1 ± 0.5	17.0 ± 2.6	21.5 ± 3.3
*M. elongata* AG77	238.8 ± 14.5	94.6 ± 4.5	42.4 ± 2.3	4.3 ± 0.5	89.1 ± 4.8
*M. gamsii* GBAus 22	178.0 ± 23.9	54.9 ± 3.9	29.3 ± 2.1	1.7 ± 0.5	66.1 ± 2.2
*M. elongata* AG77 and *N. oceanica*	168.5 ± 8.9	62.1 ± 3.0	16.3 ± 1.1	12.0 ± 0.9	46.5 ± 3.7
*M. gamsii* GBAus22 and *N. oceanica*	163.3 ± 10.5	42.0 ± 9.5	17.5 ± 1.7	9.0 ± 1.4	36.1 ± 6.1

*M. elongata* AG77/*M. gamsii* GBAus22 and *N. oceanica*, co-cultivation of the alga and fungi in *f*/2 medium for 6 days in shaker flasks. Results are the average of three biological replicates with standard deviations

*TAG* triacylglycerol, *ARA* arachidonic acid (20:4), *EPA* eicosapentaenoic acid (20:5), *PUFAs* polyunsaturated fatty acids (e.g. 18:2, 18:3, 20:4 and 20:5)

Compared to regular PDB fungal growth medium, *f*/2 medium has a high salt concentration and an elevated pH (= 7.6) and lacks sugar [36]. Thus, *M. elongata* AG77 and *M. gamsii* GBAus22 were incubated in different media in shaker flasks to test the impact on lipid metabolism of high pH (PDB medium, pH7.6), high pH and high salinity (*f*/2 + 1% sugar), and high pH and high salinity with sugar starvation (*f*/2 medium). These adverse conditions generally increased the TAG and total lipid content of *M. elongata* AG77 and *M. gamsii* GBAus22, especially under high salinity condition (PDB pH7.6 compared to *f*/2 + 1% sugar) (Additional file 1: Table S1). Compared to *M. gamsii* GBAus22, *M. elongata* AG77 showed a significant increase in TAG and total lipid under high pH (PDB, from pH 5.3 to 7.6), and a lower increase in total lipid, and slight decrease in TAG, upon sugar starvation (*f*/2 + 1% sugar compared to *f*/2) (Additional file 1: Table S1). These adverse conditions reduced the contents of ARA and total PUFAs in *M. gamsii* GBAus22, while EPA increased upon high pH but decreased under high salinity and sugar starvation (Additional file 1: Table S1). In contrast, *M. elongata* AG77 had increased contents of ARA and PUFAs in response to sugar starvation but these fatty acids decreased under high pH and high salinity conditions; EPA of *M. elongata* AG77 was decreased under all stress conditions compared to regular growth condition (Additional file 1: Table S1).

### Increasing TAG content in *N. oceanica* cells using ammonium as the N source

It has been reported that TAG is the major compound for transitory carbon storage in *N. oceanica* cells grown under light/dark cycles [37]. However, the TAG content was relatively low when cells were grown under regular conditions [13, 38]. Indeed, *N. oceanica* cells produced fewer and smaller lipid droplets than the fungi during incubation in *f*/2 medium visible in confocal micrographs (Fig. 3). To increase TAG yield in *N. oceanica*, two approaches were employed: nutrient deprivation and genetic engineering. N deprivation is one of the most efficient ways to promote TAG synthesis in microalgae [2, 7, 39]. Following 120-h N deprivation in shaker flasks, TAG accumulated in *N. oceanica* accounted for up to ~ 70% of the total lipid fraction (Additional file 2: Figure S1A), which is over 20% of DW (Additional file 2: Figure S1B). The content of TAG quickly increased following N deprivation and decreased following N resupply, indicating that *N. oceanica* cells are very sensitive to the N supply (Additional file 2: Figure S1). Under laboratory conditions, N deprivation of algal cultures can be performed by centrifugation to pellet the algal cells, followed by washes and resuspension in N-depleted medium. However, this approach is not practical during scale up for industrial purposes.

**Fig. 5:**
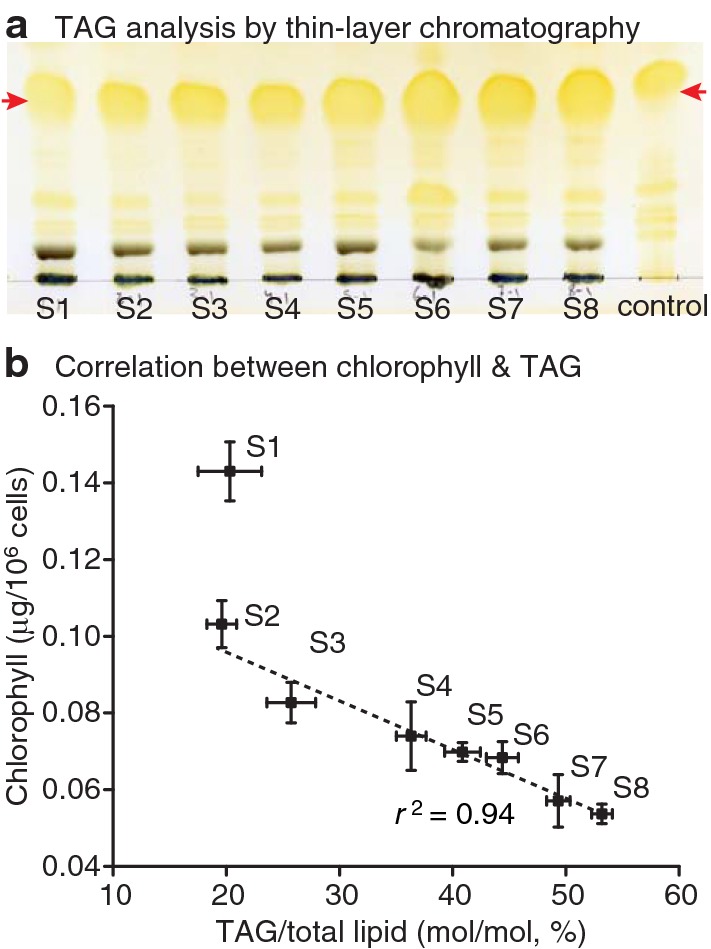
Chlorophyll as proxy of triacylglycerol accumulation. **a** Analysis of triacylglycerol (TAG) by thin layer chromatography (TLC). Algal cells were incubated in environmental photobioreactor (ePBR) containing *f*/2-NH4Cl medium after inoculation to stationary phase and then prolonged incubation. Red arrowheads indicate the TAG bands. S1 to S8, day 1 to 8 after the cells reached stationary phase; control, TAG standard. **b** Inverse correlation of chlorophyll content and TAG-to-total-lipid ratio following prolonged incubation in the ePBR containing *f*/2-NH_4_Cl medium. TAG and total lipid were subjected to transesterification and the resulting fatty acid methyl esters were quantified by GC–FID. *r*^2^, correlation coefficient; *n* = 4

Thus, we developed a limited N supply-culturing method for large-volume cultures to induce TAG accumulation largely without compromising growth and biomass yields. To mimic natural cultivation conditions for *N. oceanica*, such as an open-pond system, we used for these experiments environmental photobioreactors (ePBRs). We grew the algal cells under varying light conditions (0–2000 μmol photons m^−2^ s^−1^) under long-day (14/10 h light/dark) cycles (Additional file 3: Figure S2), and we sparged with 5% CO_2_ at 0.37 L min^−1^ for 2 min per h at 23 °C primarily to adjust the pH in the reactor vessel as previously described in [40]. Illumination in the ePBR is provided by a high-power white LED light on top of a conical culture vessel (total height of 27 cm) containing 330 mL of algal culture (20 cm in depth), which was designed to simulate pond depths from 5 to 25 cm [40]. We substituted several N sources in the *f*/2 medium for the incubation of *N. oceanica* containing set amounts of either ammonium, nitrate, or urea. Compared to nitrate and urea, *N. oceanica* grew faster in the *f*/2-NH_4_Cl medium (Additional file 4: Figure S3A), consistent with previous observations for *Nannochloropsis* sp. [41] and *Nannochloropsis gaditana* [42]. The DW of *N. oceanica* cells per L was also higher in the *f*/2-NH_4_Cl culture after 7-day incubation in the ePBR (Additional file 4: Figure S3B). Intriguingly, the cells grown in *f*/2-NH_4_Cl medium turned from vivid green to yellow following 7-day incubation once they reached stationary phase (Additional file 3: Figure S2A), indicative of chlorophyll degradation in the algal cells. Lipid analysis by TLC (Fig. 5a) and GC–FID (Fig. 5b) demonstrated TAG accumulation during the period of days from 2 to 8 after the culture reached stationary phase (incubation time S2 to S8), which was inversely correlated with chlorophyll content, while cell density and dry weight remained at similar levels during this period (Additional file 4: Figure S3C, D). Previously, to prevent carbon limitation, NaHCO_3_ was added to *N. oceanica* cultures in shaker flasks [43]. Here using the ePBRs, we noticed that the addition of NaHCO_3_ prevented acidification of the cultures, which were sparged with 5% CO_2_ (Additional file 5: Figure S4A). *N. oceanica* cells accumulated more TAG upon acidification in the culture medium without NaHCO_3_ supply, especially from S6 to S8, compared to the NaHCO_3_ culture (Additional file 5: Figure S4A, B).

### Increasing the yields of TAG and total fatty acid in alga-fungus aggregates

**Fig. 6:**
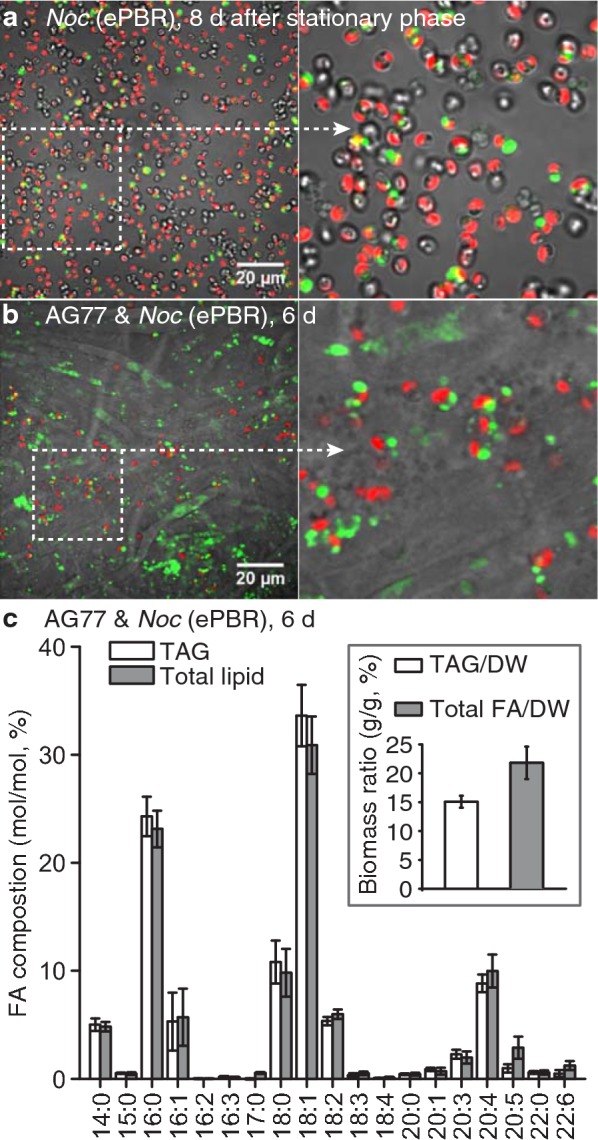
Increasing triacylglycerol (TAG) content in *N. oceanica* using limited ammonium as N source. **a** *N. oceanica* (*Noc*) cells produce large lipid droplets during prolonged incubation in the environmental photobioreactor (ePBR) containing *f*/2-NH_4_Cl medium. *Noc* cells grow fast in *f*/2-NH_4_Cl medium and suffer from nutrient limitation after 8 days in the stationary phase, when the confocal micrographs were taken. Green fluorescence indicates lipid droplets stained with BODIPY, while red fluorescence represents autofluorescence of *Noc* chloroplasts. **b** Lipid droplet staining of *M. elongata* AG77 and *Noc* cells after 6 days co-cultivation in shaker flasks following the incubation of the algae in ePBRs. **c** Fatty acid (FA) analyses of triacylglycerol and total lipid in the alga-fungus aggregate as shown in (**b**). Biomass ratio of TAG or total FA relative to the total cell dry weight (DW). *n* = 5

*Nannochloropsis oceanica* cells were inoculated to ~ 1 × 10^6^ mL^−1^ in *f*/2-NH_4_Cl medium in the ePBR and then grown to stationary phase. The cultures were further incubated for 8 days to allow the algal cells accumulate high levels of TAG as described above. Following this prolonged incubation in the ePBR, *N. oceanica* cells had a high TAG content, as shown by direct quantification (Fig. 5b) and confocal microscopy (Fig. 6a). These high-oil algal cells were then transferred from the ePBR to flasks, and ~ 3 times biomass of fungal mycelium was added. This led to the formation of algal–fungal aggregates during 6 days of co-cultivation in flask cultures. We did not attempt to co-cultivate algae and fungi in the ePBRs to avoid fungal contamination of the ePBRs and aggregation disruption by the constant stir bar motion. Following prolonged incubation in the ePBR, *N. oceanica* cells showed a high TAG content, as determined by direct quantification (Fig. 5b) and confocal microscopy (Fig. 6a). Following a subsequent co-cultivation of algal and fungal cells for 6 days, the algal–fungal aggregates were then collected by mesh filtration for further microscopy and lipid analysis. Both alga and fungus contained a high number of lipid droplets (Fig. 6b), and were enriched in TAG (~ 15% of DW) and total fatty acid (~ 22% of DW) (Fig. 6c), which was much higher than that for *f*/2 medium-grown algal cells that produced 6.2% TAG and 16.9% total fatty acid of DW (Table 1) with fungal co-cultivation. The overall oil productivity was increased from ~ 0.25 g L^−1^ day^−1^ TAG and ~ 0.67 g L^−1^ day^−1^ total fatty acid, when the algae were grown in *f*/2 medium in shaker flasks followed by co-cultivation with fungal cells, to 0.59 g L^−1^ day^−1^ TAG and 0.86 g L^−1^ day^−1^ total fatty acid, when algae were grown in *f*/2-NH_4_Cl medium in the ePBR followed by co-cultivation with fungal cells. In addition, the oil from these algal–fungal aggregates contained ~ 10% ARA in TAG and total fatty acid (Fig. 6c).

Genetic engineering was also performed to increase TAG content in *N. oceanica* by overexpressing *DGTT5* under the control of a strong elongation factor gene (*EF*) promoter (Additional file 6: Figure S5). Two strains (*DGTT5ox3* and *ox6*) obtained from nuclear transformation displayed a strong increase in the *DGTT5* expression compared to the wild-type and empty-vector control by quantitative RT-PCR (Additional file 7: Figure S6A). The construct generated DGTT5 fused to the cerulean fluorescent protein, and the presence of the cerulean protein in *DGTT5ox3* and *ox6* was confirmed by confocal microscopy (Additional file 7: Figure S6B). Subsequent BODIPY staining showed that the *DGTT5oxs* lines had more lipid droplets compared to the wild type under regular growth condition (Additional file 7: Figure S6C), which was further confirmed by quantitative lipid analysis (Additional file 7: Figure S6C). Because the elongation factor promotor was inhibited by N starvation, the *DGTToxs* cells were not incubated in *f*/2-NH_4_Cl medium but grown in regular *f*/2 medium, and then log-phase cells of *DGTT5oxs* were co-cultivated with ~ 3 times biomass of *M. elongata* AG77, and TAG yields of the algal–fungal aggregates were determined. The *DGTT5oxs* had increased TAG content (~ 10% of DW) compared to the wild type (~ 6% of DW) (Additional file 7: Figure S6E), which was less productive than the cells cultivated in a low N-medium (~ 15% of DW).

### Fatty acid and TAG synthesis pathways in *M. elongata* AG77

**Fig. 7:**
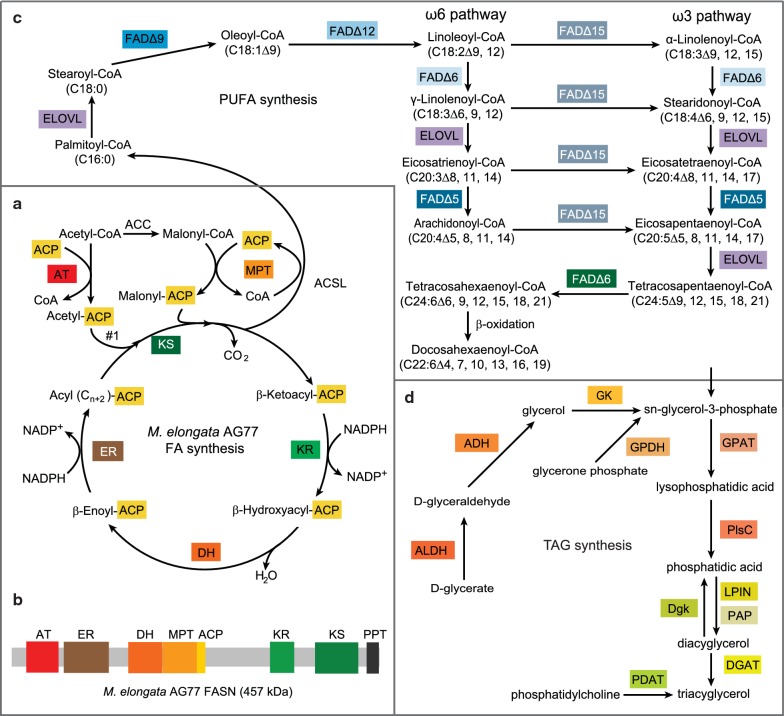
Predicted fatty acid/lipid pathways in *M. elongata* AG77. Proteins likely involved in the synthesis of fatty acids (FA), polyunsaturated fatty acids (PUFA), and triacylglycerol (TAG) are identified in the sequenced genome of *M. elongata* AG77 at the JGI fungal genome portal MycoCosm (Additional file 8: Table S2). **a** FA synthesis. ACP, acyl carrier protein; AT, acetyltransferase; MPT, malonyl/palmitoyl transferase; ACSL, acyl-CoA synthetase; KS, β-ketoacyl synthase; ER, β-enoyl reductase; DH, dehydratase; KR, β-ketoacyl reductase. **b** Linear domain organization of fatty acid synthase (FASN) of *M. elongata* AG77. PPT, phosphopantetheine transferase. **c** PUFA synthesis. ELOVL, fatty acid elongase; FAD, fatty acid desaturase. Fatty acids are designated by the number of total carbon: the number of double bonds. The position of specific double bonds is indicated either from the carboxyl end (∆) or from the methyl end (*ω*). **d** TAG synthesis. ALDH, aldehyde dehydrogenase; ADH, alcohol dehydrogenase; GK, glycerol kinase; GPDH, glycerol-3-phosphate dehydrogenase; GPAT, glycero-3-phosphate acyltransferase; PlsC, 1-acyl-*sn*-glycerol-3-phosphate acyltransferase; LPIN, phosphatidate phosphatase LPIN; PAP, phosphatidate phosphatase 2; Dgk, diacylglycerol kinase; DGAT, diacylglycerol acyltransferase; PDAT, phospholipid diacylglycerol acyltransferase

The genome of *N. oceanica* CCMP1779 has been sequenced and analyzed for the presence of metabolic pathway genes for PUFA and TAG biosynthesis [13], information used in the genetic engineering for increased EPA content [18]. For *Mortierella* fungi, nuclear transformation methods were established [44, 45], and the *M. elongata* AG77 genome has been sequenced and annotated [32], but lipid metabolic pathways have not yet been reconstructed. Thus, we applied the genome browser and BLAST tools from the JGI fungal genome portal MycoCosm to predict fatty acid, PUFA, and TAG synthesis pathways for *M. elongata* AG77. The fatty acid synthesis pathway (Fig. 7a) was predicted according to gene candidates (Additional file 8: Table S2) and previous reports on eukaryotic fatty acid pathways [46, 47]. *M. elongata* AG77 has a type-I fatty acid synthase with a similar domain organization as found in yeast (Fig. 7b) [46]. Nine elongases and twelve desaturases were identified within the *M. elongata* AG77 genome for PUFA synthesis, including a ∆15 fatty acid desaturase (FAD) for EPA synthesis (Fig. 7c, Additional file 8: Table S2). Three DGATs and one PDAT (phospholipid:diacylglycerol acyltransferase) were present in the *M. elongata* AG77 genome, which is similar to what was reported for *M. alpina* [47].

## Discussion

Microalgal biofuel is a renewable and sustainable energy source that can be a supplement or alternative to fossil fuels [1, 2, 26]. Although microalgal biofuel has many competitive advantages, current high costs associated with algal biofuel production limit its commercialization [20–22]. To overcome the major challenges in algal biofuel production, including high costs of harvesting, lipid extraction, and nutrient supply, as well as low oil content in algae, we developed a method to harvest the oleaginous marine alga *N. oceanica* through bio-flocculation with an oleaginous fungus *M. elongata* AG77. By optimizing the incubation conditions, we have increased TAG biofuel yields.

### Bio-flocculation of *N. oceanica* by *Mortierella* fungi and physical interactions between the partners

Physical interaction and symbioses between fungi and algae naturally occur, such as in lichens, where autotrophic algae co-exist with fungi and develop a mutualistic relationship [48]. In fact, about one-fifth of all the known extant fungal species are symbiotically associated with photobionts such as algae and cyanobacteria in lichenized forms [49]. Taking advantage of known algal–fungal interactions, filamentous fungi have been tested for harvesting microalgae in recent years [23, 29]. However, although some of the tested fungal strains including *Aspergillus nomius* and *A. fumigatus* can very efficiently flocculate algae, they are not enriched in oil such that they dilute the algal oil in the resulting biomass [50, 51]. In addition, some fungal strains reported to flocculate algae such as *A. fumigatus* and *M. circinelloides* are animal pathogens and can threaten human health [28, 30].

In this study, we sought to use nonpathogenic oleaginous fungi to test their potential for bio-flocculation of microalgae. Among the screened fungi, *M. elongata* AG77 can be used to efficiently harvest *N. oceanica* cells (Fig. 1a). *M. elongata* AG77, which grows in soil and associates with plant roots [52], produces TAG and PUFAs including ARA and EPA (Table 1). *N. oceanica* cells were captured by the *M. elongata* AG77 mycelium. With the addition of ~ threefold algal biomass, *M. elongata* AG77 can flocculate ~ 65% of algal cells after 6-day co-cultivation in flask cultures and was the most efficient isolate tested (Fig. 1c, e). Other studies have shown high efficiency of algal flocculation by fungi [30, 50, 51]. However, the exact nature of the interaction between algae and fungi remains to be determined. Previous studies have suggested that the fungal cell wall has a positive charge that likely attracts and neutralizes the negatively charged algal cells [30, 50, 51], similar to chemical flocculation methods using metal salts and cationic polymers. Fungal cell walls contain chitin, which has a strong positive charge, and the algal flocculation efficiency was correlated with the chitin content (12–16% of DW) in the five fungal strains tested for harvesting *Nannochloropsis* sp. [50]. However, zeta potential measurements that represent the surface charges do not necessarily correlate with flocculation efficiency [30, 50], suggesting that the flocculation of algae by fungi is not only dependent on the ionic attraction. In this study, we investigated details of the physical interaction between *N. oceanica* and fungi with SEM. Micrographs show *N. oceanica* cells trapped within the mesh-like mycelium of *M. elongata* AG77 and NVP64 (Fig. 2a, b). High-magnification images (×20,000 to ×250,000) show that *N. oceanica* has extensions along the outer layer of the cell wall, which appear to attach algal cells to the rugged surface of the fungal cell wall. Similar extension structures have also been observed in *N. gaditana* cells, which are connected to a thin algaenan layer of the *N. gaditana* cell wall, based on quick-freeze deep-etch EM and analyses of cell wall compositions [33]. Intriguingly, some fiber-like extensions from the *N. oceanica* cell walls seem to fuse with the surface of the fungal cell wall and form irregular tube-like structures that appear to anchor the algal cells to the mycelium (Fig. 2a, b). However, the *Morchella* strain 3668S that cannot flocculate *N. oceanica*, is also not able to capture *N. oceanica* cells in its mycelium (Fig. 2c). Considering that both *Mortierella* and *Morchella* fungi have high chitin contents in their cell wall [53, 54], *M. elongata* AG77 and NVP64 and *M. americana* 3668S likely have positive surface charges. A major physical difference between the strains is that *M. americana* 3668S has much thicker hyphae than the *M*. *elongata* strains, suggesting that the size and structure of the mycelium may be important for the physical attraction of algal cells, but fungal exudates and chemotaxis could also underlie efficient bio-flocculation.

### TAG and PUFAs are increased in *Mortierella* grown in *f*/2 medium

The oleaginous fungi tested in this study produce a large number of lipid droplets, when incubated in regular PDB medium in flaks cultures as shown by confocal microscopy (Fig. 3a–d). To flocculate *N. oceanica* cells, fungal mycelium was added into the algal culture that contains high concentration of sea salt at pH 7.6 but lacks a sugar supply. The combined stress conditions presented by *f*/2 medium elevated the TAG and PUFA contents in *M. elongata* AG77 (Additional file 1: Table S1) and decreased its growth, such that it would not outgrow the algal cells. In fact, *N. oceanica* cells had normal diameter (Fig. 1f) and low amount of oil when co-cultivated with the fungi (Fig. 3f–i), similar to the cells that were grown alone and those grown in *f*/2 medium (Fig. 3e).

*Mortierella elongata* AG77 is a representative strain of *Mortierella*, a widespread genus of soil fungi, which are industrially important for the production of ARA-rich oil [34, 35]. *Mortierella* fungi grow fast in nutrient-rich media, and studies have shown that *M. elongata* CBS 121.71 and *M. alpina* SD003 can also grow in wastewater [55, 56], while a related species *Umbelopsis isabellina* can grow on sewage sludge [57], suggesting low-cost incubation methods of growing *M. elongata* AG77 for the bio-flocculation of algae.

### Increasing TAG in *N. oceanica* cells through genetic engineering and by optimizing incubation conditions

In recent years, intensive studies have been performed on *Nannochloropsis* because of its potential for biofuel production [7, 10–12]. Besides its high TAG content, *Nannochloropsis* is enriched in PUFAs such as EPA, a high-value omega-3 fatty acid [18]. However, *Nannochloropsis* species, such as *N. oceanica*, do not have high oil content under favorable growth conditions (Fig. 3e; Additional file 2: Figure S1) [13, 37]. Thus, although the *N. oceanica*–*M. elongata* AG77 co-culture generated favorable yields of ARA and EPA, TAG production was only ~ 6% of DW—lower than the ~ 10% TAG yield when using *M. elongata* AG77 alone (Table 1, Fig. 4). *Nannochloropsis* accumulates high levels of TAG and total fatty acids in response to stress conditions such as N deprivation (Additional file 2: Figure S1) and following the addition of the ribosome inhibitor cycloheximide [8]. Similarly, TAG and total lipid stimulation by environmental stresses have been commonly observed in the other microalgae such as green algae and diatoms [2, 7]. TAG is usually used as the feedstock for biodiesel and can be converted into high-energy fatty acid methyl esters (FAMEs) by transesterification with glycerol as a valuable byproduct [25, 58]. In addition, TAG is a nonpolar lipid stored in lipid droplets and is more readily extractable with organic solvent than the polar membrane lipids [59]. Thus, the increasing TAG content can simplify the lipid extraction and transesterification, thereby reducing the cost of processing. To increase TAG content in *N. oceanica*, we developed an N starvation method for *N. oceanica* using ammonium as the N source. We tested the growth of *N. oceanica* with three N sources—ammonium, nitrate, and urea—in ePBRs that can mimic natural growth conditions such as open-pond system (Additional file 3: Figure S2) [40]. *N. oceanica* cells grow significantly faster and have higher biomass in the ammonium medium than the nitrate and urea media, following the same period of incubation (Additional file 4: Figure S3). In addition, *N. oceanica* cells consumed ammonium faster than nitrate and approached N starvation during the week after the culture reached stationary phase (Fig. 5; Additional file 3: Figure S2A), consistent with the previous observation that *Nannochloropsis* sp. grow faster in ammonium medium with a significantly higher uptake rate of ammonium than nitrate [41]. N deprivation is one of the most efficient ways to stimulate TAG accumulation in microalgae. However, it is not feasible to centrifuge the cells and change the medium at large industrial scales, as one can do in the laboratory. Considering that *N. oceanica* cells are very sensitive to the N supply (Additional file 2: Figure S1), feeding ammonium to the culture with limited amount of N, or in multiple-low doses, can allow for rapid cell growth, and provide a means to control the abundance of N in the culture. The N supply can be stopped as the culture reaches the stationary phase such that the cells exhaust their N supply and enter N starvation, while the total cell count and biomass are not compromised (Additional file 4: Figure S3C, D).

We have developed a quick and simple method to indirectly monitor TAG content for *N. oceanica* using chlorophyll as an indicator. Chlorophyll content is inversely correlated with TAG content during nutrient deprivation (Fig. 5b). In fact, thylakoid membrane degradation, in parallel with TAG accumulation, has been commonly observed in microalgae and plant vegetative tissues. These combined processes can reduce photosynthesis and photooxidative stress, sequester toxic compounds such as free fatty acids, and save the acyl chains for later resynthesis of membranes when conditions improve [7, 39, 60].

We also noticed that culture acidification caused by CO_2_ supply led to faster and higher TAG accumulation in *N. oceanica* cells (Additional file 5: Figure S4). The described N starvation method should be applicable for most microalgal species, especially for the wild-type and transgenic strains optimized for growth rate, biomass, and stress resistance, but which have low oil content under regular growth condition. Under the right conditions, TAG accumulation occurs once the culture reaches stationary phase/high biomass. The N starvation method we describe will not reduce cell growth or biomass, which has been commonly observed for genetically engineered high TAG strains [8, 9, 19]. On the contrary, the higher the biomass and cell concentration, the faster the algal cells deplete their N source and generate more TAG. Taking advantage of the high TAG-yielding *N. oceanica* cells produced by the N starvation method (Fig. 6b), we obtained high yields of TAG (~ 15% of DW) and total fatty acids (~ 22% of DW) in the *N. oceanica*-AG77 aggregate (Fig. 6b, c).

### Potential of genetic engineering of PUFAs in *N. oceanica* and *M. elongata* AG77

Both *N. oceanica* CCMP1779 and *M. elongata* AG77 generate ARA and EPA: ARA is low in *N. oceanica* but high in AG77, whereas EPA is high in *N. oceanica* but low in AG77 (Fig. 4a, b). Besides ARA and EPA, *M. elongata* AG77 also produces ~ 5% DHA (Fig. 4b). Thus, the complementary production of ARA, EPA, and DHA, high-value products with important health benefits [61], could increase the value of the oil produced by aggregates of *N. oceanica* and *M. elongata* AG77. In addition, the genome of *N. oceanica* CCMP1779 and its BLAST and gene browser tools are available [13], and genetic engineering methods such as CRISPR–Cas9 and overexpression systems have been established for *N. oceanica* [9, 16, 18]. Recent studies have successfully increased the EPA production by the overexpression of ∆5 and ∆12 FADs in *N. oceanica* [18] and induced TAG production under normal growth condition by overexpressing *N. oceanica DGTT5* [9].

Transformation methods for *Mortierella* fungi such as *M. alpina* have been developed [45, 62], and the genome of *M. elongata* AG77 is available [32]. In this study, we analyzed the genome data and predicted the PUFA and TAG synthesis pathways (Fig. 7), based on the gene candidates involved in the processes (Additional file 8: Table S2), which will facilitate genetic engineering on PUFA and TAG products in *M. elongata* AG77 in the future.

## Conclusions

*Nannochloropsis oceanica* CCMP1779 and *M. elongata* AG77 are model species of algae and fungi, respectively. Both genera contain many other oleaginous strains (e.g., *N. gaditana* and *M. alpina*). The bio-flocculation method demonstrated in this study could be performed with other strains leading to synergistic increases in TAG content of the biomass. The N starvation and seawater medium can also be used on transgenic strains with high TAG or high biomass traits, which has the potential to increase oil yield and reduce the cost of oil extraction and transesterification. *N. oceanica* grows in seawater, and *M. elongata* AG77 can be incubated with wastewater and sewage that most likely can reduce the cost of nutrient supply. Overall, the combination of *N. oceanica and M. elongata* AG77 has the potential to overcome some of the barriers for the commercialization of microalgal/fungal biofuels and biomaterials.

## Methods

### Materials and growth condition

The marine alga *Nannochloropsis oceanica* CCMP1779 was obtained from the Provasoli-Guillard National Center for Culture of Marine Phytoplankton. *N. oceanica DGTT5*-overexpressing strains *DGTT5ox*3 and *DGTT5ox*6 were generated and examined by quantitative RT-PCR as previously described [9]. The alga was grown in *f*/2 medium containing 2.5 mM NaNO_3_, 0.036 mM NaH_2_PO_4_, 0.106 mM Na_2_SiO_3_, 0.012 mM FeCl_3_, 0.012 mM Na_2_EDTA, 0.039 μM CuSO_4_, 0.026 μM Na_2_MoO_4_, 0.077 μM ZnSO_4_, 0.042 μM CoCl_2_, 0.91 μM MnCl_2_, 0.3 μM thiamine HCl/vitamin B1, 2.05 nM biotin, 0.37 nM cyanocobalamin/vitamin B12 [63], and 20 mM sodium bicarbonate and 15 mM Tris buffer (pH 7.6) to prevent carbon limitation [43]. The cells were grown in batch cultures in two systems: shaker flask with *f*/2 medium (under ~ 80 μmole photons m^−2^ s^−1^ at 23 °C) or in ePBRs [40] with *f*/2-NH_4_Cl (2.5 mM NH_4_Cl replacing 2.5 mM NaNO_3_) or *f*/2-urea (2.5 mM urea replacing 2.5 mM NaNO_3_) medium with varying light conditions as indicated (Additional file 3: Figure S2, 0–2000 μmol photons m^−2^ s^−1^ under diurnal 14/10 h light/dark cycle) at 23 °C and sparged with air enriched to 5% CO_2_ at 0.37 L min^−1^ for 2 min per h. For prolonged-incubation in the ePBR, *N. oceanica* cells were inoculated to ~ 1 × 10^6^ mL^−1^ in *f*/2-NH_4_Cl medium and grown to stationary phase. The cultures were further incubated for 8 days to increase TAG content.

*Mortierella* fungi *M. elongata* AG77, *M. elongata* NVP64, and *M. gamsii* GBAus22 isolates were isolated from soil samples collected in North Carolina (AG77), Michigan (NVP64), USA, and Australia (GBAus22). *Morchella americana* 3668S was obtained from the USDA NRRL Agriculture Research Station. Fungal samples were incubated in PDB medium (12 g L^−1^ potato dextrose broth and 1 g L^−1^ yeast extract, pH5.3) at 23 °C. For the algal–fungal co-cultivation, fungal mycelia were briefly blended into small pieces (~ 1 cm) with a sterilized blender and were collected by centrifugation (3000*g* for 3 min) after 24-h recovery in PDB medium. The samples were washed twice with *f*/2 or *f*/2—NH_4_Cl medium and resuspended in 5–10 mL of the respective medium. One-third of the samples were used for determining dry biomass: 1 mL culture was transferred and filtered with pre-dried and -weighed Whatman GF/C filters and dried overnight at 80 °C. The remaining fungal mycelia were added to the *N. oceanica* culture (~ 3 times to algal biomass) for 6-day co-cultivation on a shaker (~ 60 rpm) under continuous light (~ 80 μmol photons m^−2^ s^−1^) at 23 °C. Cell size and concentration of *N. oceanica* cultures were determined with a Z2 Coulter Counter (Beckman). The bio-flocculation efficiency of *N. oceanica* cells using fungal mycelium was determined by the cell density of uncaptured algal cells compared to that of an algal culture control, to which no fungus was added.

### Light microscopy

Interactions between the algal and fungal cells were examined by light microscopy using an inverted microscope with DIC function (DMi8, Leica). DIC images were taken of the algae–fungi aggregates after 6-day co-cultivation.

### Scanning electron microscopy

Scanning electron microscopy was performed to investigate the physical interaction between *N. oceanica* and fungi at the Center for Advanced Microscopy of Michigan State University (CAM, MSU). Algae–fungi aggregates were collected after 6-day co-culture of the alga *N. oceanica* with *M. elongata* (AG77 and NVP64) or *M. americana* 3668S and were fixed in 4% (v/v) glutaraldehyde solution, followed by drying in a critical point dryer (Model 010, Balzers Union). The samples were then mounted on aluminum stubs with high vacuum carbon tabs (SPI Supplies), and were coated with osmium using a NEOC-AT osmium coater (Meiwafosis). The samples were observed with a JSM-7500F scanning electron microscope (Japan Electron Optics Laboratories).

### Confocal microscopy

Confocal microscopy was carried out to visualize and briefly quantify lipid droplets in the alga and the fungi. The samples were stained with 10 μg mL^−1^ BODIPY 493/503 (ThermoFisher Scientific) in PBS buffer for ~ 30 min at 23 °C. After two washes with PBS buffer, the samples were observed using an Olympus Spectral FV1000 microscope at CAM, MSU. An argon (488 nm) laser and a solid-state laser (556 nm) were used for BODIPY (emission, 510–530 nm) and chloroplast (emission, 655–755 nm) fluorescence. *N. oceanica* DGTT5 fused to the cerulean fluorescent protein was overproduced using the EF promotor as previously published [9]. The presence of the fluorescent protein in the *DGTT5ox* strains was detected by confocal microscopy (emission 420–440 nm) using a LSM 510 Meta Confocal Laser Scanning Microscope (Zeiss).

### Lipid extraction and analysis

For lipid extraction, log-phase *N. oceanica* cells grown in *f*/2 medium were collected by centrifugation (4000*g* for 5 min). To test lipid content in different media, *Mortierella* fungi grown in PDB medium were washed twice with different media: PDB medium, pH7.6; *f*/2 medium with 1% glucose; *f*/2 medium. The cells were incubated in the respective medium for 48 h and were subsequently collected for lipid extraction by centrifugation (3000*g* for 3 min). For total lipid extraction, algae–fungi aggregates were collected by mesh filtration and frozen in liquid nitrogen prior to grinding with mortar and pestle. The fine powders were transferred to a pre-weighed and -frozen glass tube, and total lipids were extracted with methanol-chloroform-88% formic acid (1:2:0.1 by volume) on a multi-tube vortexer (1500*g* for ~ 20 min; Benchmark Scientific), followed by addition of 0.5 volume of 1 M KCl and 0.2 M H_3_PO_4_. After phase separation by centrifugation (2000*g* for 3 min), total lipids were collected for TAG separation and fatty acid analysis. The solids were dried at 80 °C overnight to provide the nonlipid biomass.

TAG was separated by TLC using G60 silica gel TLC plates (Machery-Nagel) developed with petroleum ether-diethyl ether-acetic acid (80:20:1 by volume). An internal standard of 5 μg of tridecanoic acid (C13:0) or pentadecanoic acid (C15:0) was added to each tube containing TAG or total lipid. FAMEs were then prepared with 1 M methanolic HCl at 80 °C for 25 min, and were phase separated with hexane and 0.9% NaCl and nitrogen-dried and resuspended in ~ 50 μL of hexane. Gas chromatography and flame ionization detection (Agilent) were used to quantify the FAMEs in TAG and total lipid as previously described [64]. Dry weight of algae–fungi biomass was obtained by summing up nonlipid and total lipid mass.

### Chlorophyll measurement

*Nannochloropsis oceanica* cells were collected by centrifugation from 1 mL culture aliquots during prolonged incubation in the ePBRs. Chlorophyll of the pelleted cells was extracted with 900 μL of acetone:DMSO (3:2, v/v) for 20 min with agitation at 23 °C, and measured with an Uvikon 930 spectrophotometer (Kontron) [60].

### Generation of *N. oceanica DGTT5* overexpression strains

The NoDGTT5 sequence was amplified by PCR using the primers: forward, 5′-ATGACGCCGCAAGCCGACATCACCAGCAAGACGA-3′; reverse, 5′-CTCAATGGACAACGGGCGCGTCTCCCACTCC-3′, followed by gel purification with E.Z.N.A. Gel Extraction Kit (OMEGA Biotek) and ligated into the pnoc ox cerulean hyg vector (Additional file 6: Figure S5A) to obtain the final vector pnoc ox DGTT5 cerulean hyg (Additional file 6: Figure S5B) for nuclear transformation. *N. oceanica* CCMP1779 cells were transformed by electroporation as previously described [13].

### Prediction of fatty acid and TAG pathways

The sequenced genome of *M. elongata* AG77 [32] was annotated for genes and proteins likely involved in the synthesis of fatty acids, PUFAs, and TAGs using by BLAST searches against KOG and KEGG databases at the JGI fungal genome portal MycoCosm *M. elongata* AG77 v2.0 and by comparison to previously published annotations of lipid pathways of *Mortierella alpina* [47].

### Accession numbers

Sequence data presented in this article can be found in the genome of *N. oceanica* CCMP1779 at the JGI database (https://genome.jgi.doe.gov/Nanoce1779/Nanoce1779.home.html). Gene ID of *N. oceanica* DGTT5, CCMP1779_3915; Accession Number, KY273672. Sequence data of *M. elongata* AG77 can be found at the JGI fungal genome portal MycoCosm (https://genome.jgi.doe.gov/Morel2/Morel2.home.html). Transcript IDs and protein IDs of the genes are listed in the Additional file 8: Table S2.

## Additional files

**Additional file 1: Table S1.** Lipid and fatty acid contents of *Mortierella* fungi incubated in different media (mg g^−1^ total dry weight).

**Additional file 2: Figure S1.** Triacylglycerol content in *N. oceanica* cells.

**Additional file 3: Figure S2.** Incubation of *N. oceanica* cells in the environmental photobioreactor (ePBR).

**Additional file 4: Figure S3.** Cell growth and biomass in the environmental photobioreactor (ePBR).

**Additional file 5: Figure S4.** Triacylglycerol accumulation during prolonged-incubation in f/2 medium supplemented with or without sodium bicarbonate.

**Additional file 6: Figure S5.** Maps of the plasmids used for the generation of *N. oceanica DGTT5*-overexpressing strains.

**Additional file 7: Figure S6.** Increasing triacylglycerol content in *N. oceanica* by the overexpression of *N. oceanica DGTT5* encoding acyl-CoA:diacylglycerol acyltransferase DGTT5.

**Additional file 8: Table S2.** Predicted genes and proteins involved in fatty acid and glycerolipid synthesis in *M. elongata* AG77.

## Abbreviations

ARA: arachidonic acid
*DGTT5*: a gene encoding the type II acyl-CoA:diacylglycerol acyltransferase 5
DHA: docosahexaenoic acid
DW: dry weight
*EF*: elongation factor gene
EPA: eicosapentenoic acid
ePBR: environmental photobioreactor
FAMEs: fatty acid methyl esters
GC–FID: gas chromatography and flame ionization detection
PDAT: phospholipid:diacylglycerol acyltransferase
PDB: potato dextrose broth
PUFAs: polyunsaturated fatty acids
S2 to S8: days 2 to 8 after the culture reached stationary phase
SEM: scanning electron microscopy
TAG: triacylglycerol
TLC: thin layer chromatography
