# Supplementary material for: Enhancing oil production and harvest by combining the marine alga Nannochloropsis oceanica and the oleaginous fungus Mortierella elongata
Source: Biotechnol Biofuels. 2018 Jun 22;11:174. doi: 10.1186/s13068-018-1172-2 (PMC6013958; doi:10.1186/s13068-018-1172-2)
Supplement: Supplementary file 1 — Additional file 1: Table S1. Lipid and fatty acid contents of Mortierella fungi incubated in different media (mg g−1 total dry weight). [file 13068_2018_1172_MOESM1_ESM.pdf]

Table S1. Lipid and fatty acid contents of *Mortierella* fungi incubated in different media in shaker flasks (mg g^{-1} total dry weight).

Strains	Total lipid	TAG	ARA	EPA	PUFAs
M. elongata AG77, PDB, pH5.3	128.2 ± 11.9	15.3 ± 1.0	27.9 ± 1.3	6.14 ± 0.8	78.9 ± 1.3
M. elongata AG77, PDB, pH7.6	170.2 ± 17.6	31.8 ± 2.0	25.2 ± 3.1	1.7 ± 1.1	48.9 ± 2.9
M. elongata AG77, f/2+1% sugar	233.2 ± 21.8	106.1 ± 12.3	15.5 ± 0.2	3.0 ± 0.1	41.5 ± 1.1
M. elongata AG77, f/2	238.8 ± 14.5	94.6 ± 4.5	42.4 ± 2.3	4.3 ± 0.5	89.1 ± 4.8
M. gamsii GBAus22, PDB, pH5.3	101.2 ± 13.6	5.3 ± 1.4	33.8 ± 2.4	2.09 ± 0.08	69.9 ± 0.9
M. gamsii GBAus22, PDB, pH7.6	108.9 ± 12.5	11.7 ± 1.4	31.7 ± 1.4	2.9 ± 0.2	58.3 ± 1.8
M. gamsii GBAus22, f/2+1% sugar	139.4 ± 12.5	34.7 ± 4.4	16.4 ± 1.6	2.1 ± 0.2	39.0 ± 3.1
M. gamsii GBAus 22, f/2	178.0 ± 23.9	54.9 ± 3.9	29.3 ± 2.1	1.7 ± 0.5	66.1 ± 2.2

TAG, triacylglycerol; ARA, arachidonic acid (20:4); EPA, eicosapentaenoic acid (20:5); PUFAs, polyunsaturated fatty acids; f/2+1% sugar, f/2 medium supplemented with 1% glucose, pH7.6. Results are the average of five biological replicates with error bars indicating standard deviations.
