# Supplementary material for: Enhancing oil production and harvest by combining the marine alga Nannochloropsis oceanica and the oleaginous fungus Mortierella elongata
Source: Biotechnol Biofuels. 2018 Jun 22;11:174. doi: 10.1186/s13068-018-1172-2 (PMC6013958; doi:10.1186/s13068-018-1172-2)
Supplement: Supplementary file 2 — Additional file 2: Figure S1. Triacylglycerol content in N. oceanica cells. [file 13068_2018_1172_MOESM2_ESM.pdf]

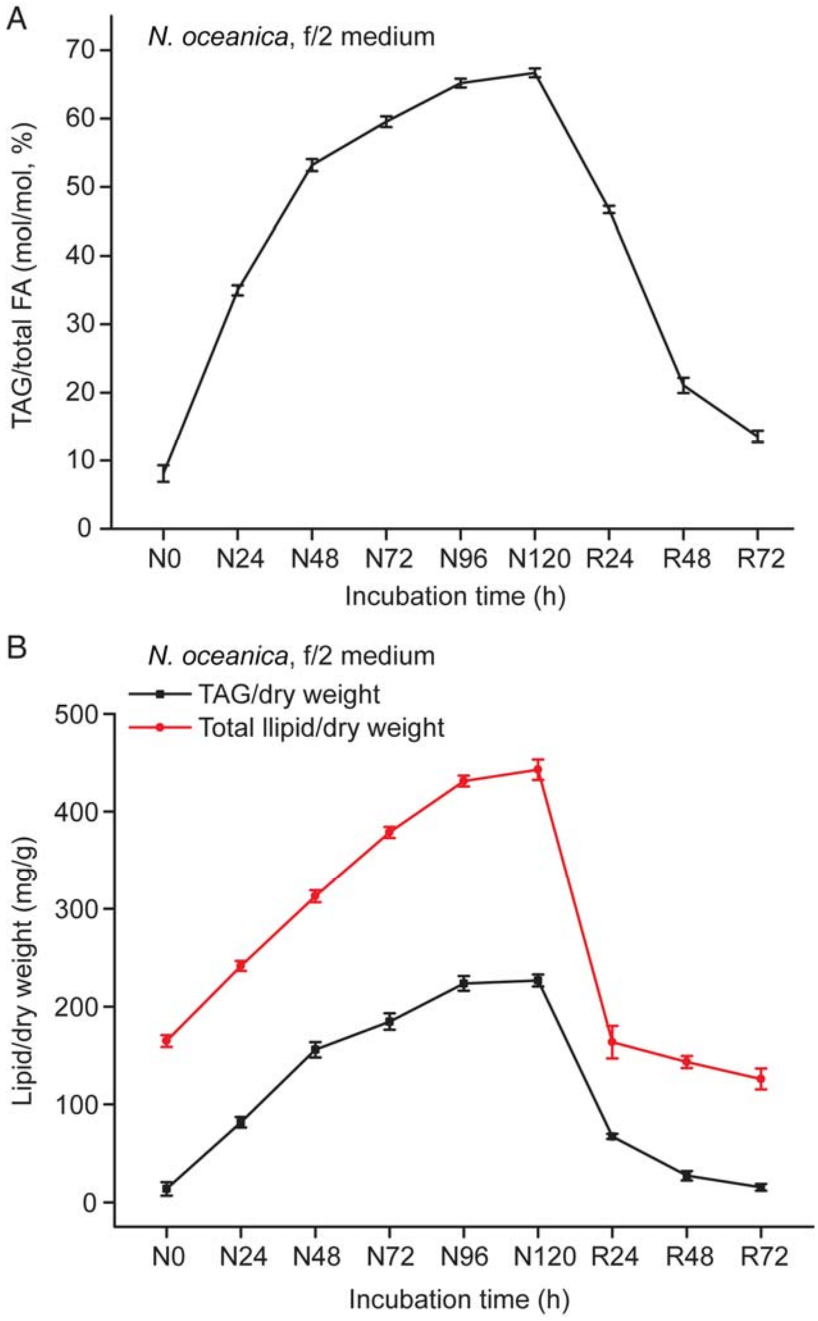

Figure S1. Triacylglycerol content in *N. oceanica* cells. A, Mole ratio of triacylglycerol (TAG) compared to total lipid. Cells were grown in shaker flasks. N0-120, N deprivation(f/2 medium lacking N 0-120 h; R24-72, N resupply (f/2) medium for 24-72 h. The average of three biological replicates and standard deviation are shown (n = 3). B, TAG and total lipid content per gram of whole cell dry weight. n = 3.
