# Supplementary material for: Enhancing oil production and harvest by combining the marine alga Nannochloropsis oceanica and the oleaginous fungus Mortierella elongata
Source: Biotechnol Biofuels. 2018 Jun 22;11:174. doi: 10.1186/s13068-018-1172-2 (PMC6013958; doi:10.1186/s13068-018-1172-2)
Supplement: Supplementary file 3 — Additional file 3: Figure S2. Incubation of N. oceanica cells in the environmental photobioreactor (ePBR). [file 13068_2018_1172_MOESM3_ESM.pdf]

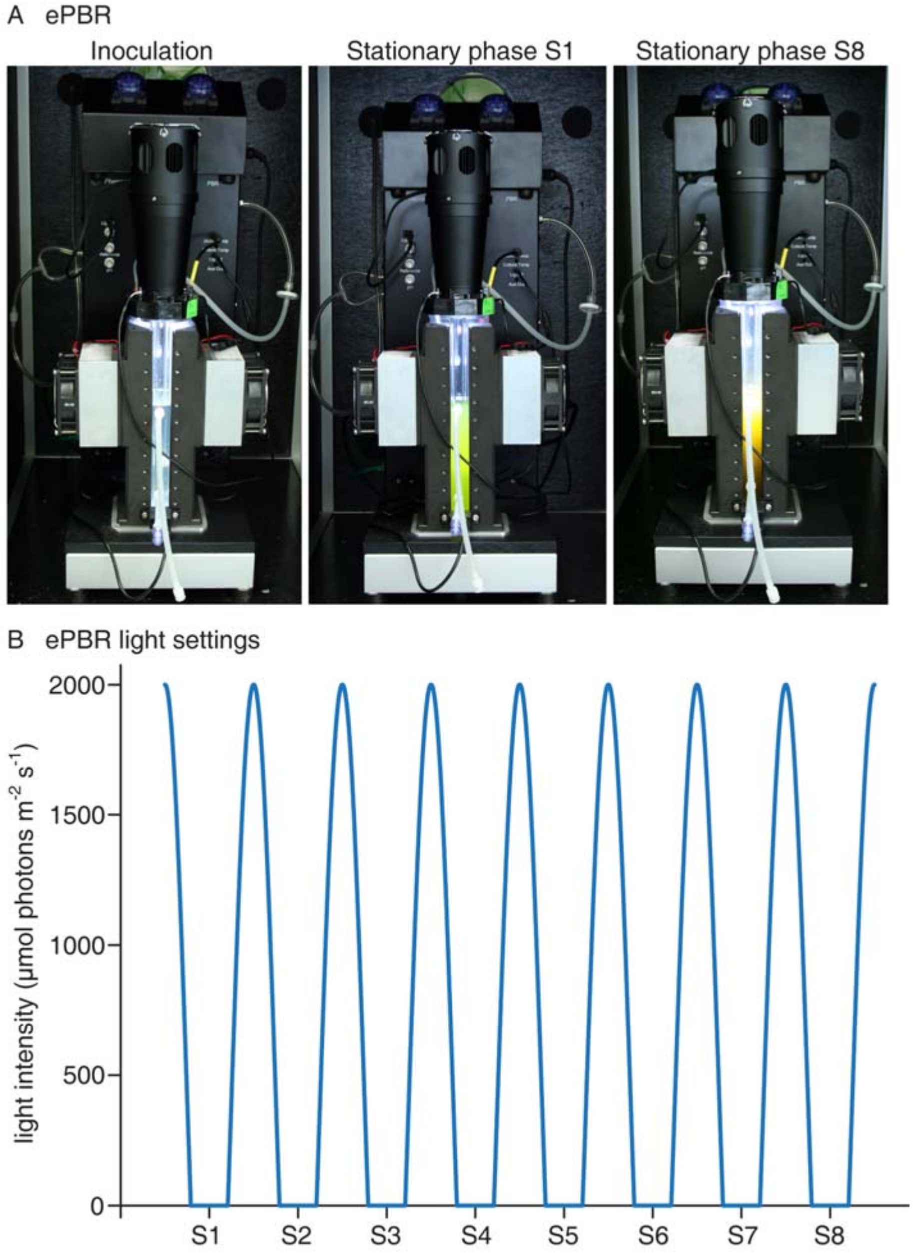

Figure S2. Incubation of *N. oceanica* cells in the environmental photobioreactor (ePBR). A, *N. oceanica* cells were inoculated in the f/2 medium containing NH_4Cl (left image) and were incubated in the ePBR to stationary phase (day 1, S1; middle image) and further grown for 8 days (S8; right image). Cultures were incubated under fluctuating light at 23°C and were sparged with air enriched to 5% CO_2 at 0.37 L min^{-1} for 2 min per h. B, Light conditions for the cultures in the ePBR: fluctuating lights (0 to 2,000 µmol photons m^{-2} s^{-1}) under diurnal 14/10 h light/dark cycle.
