# Supplementary material for: Enhancing oil production and harvest by combining the marine alga Nannochloropsis oceanica and the oleaginous fungus Mortierella elongata
Source: Biotechnol Biofuels. 2018 Jun 22;11:174. doi: 10.1186/s13068-018-1172-2 (PMC6013958; doi:10.1186/s13068-018-1172-2)
Supplement: Supplementary file 4 — Additional file 4: Figure S3. Cell growth and biomass in the environmental photobioreactor (ePBR). [file 13068_2018_1172_MOESM4_ESM.pdf]

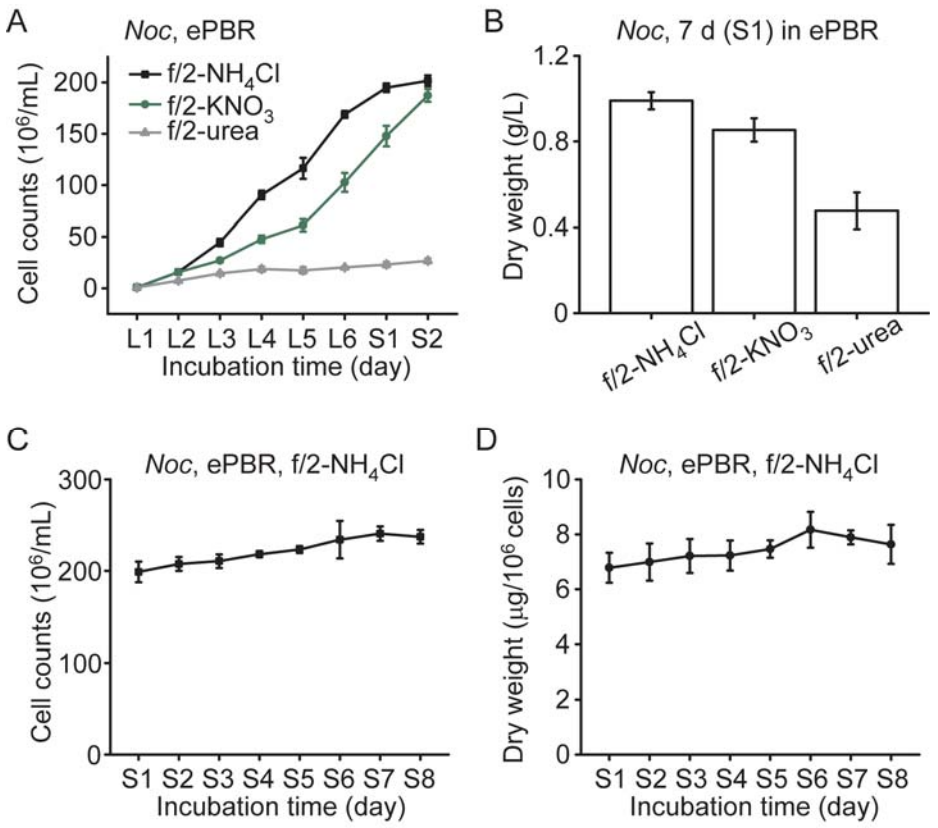

Figure S3. Cell growth and biomass in the environmental photobioreactor (ePBR). A, *N. oceanica* (*Noc*) cells were inoculated to ~1 x 10^6 mL^{-1} and incubated in the environmental photobioreactor containing modified f/2 media with NH_4Cl, KNO_3, or urea as N source. L1-6, day 1-6 of log phase; S1 and 2, day 1 and 2 of stationary phase. The average of three biological replicates and standard deviation are shown (n = 3). B, Dry weight per L of cells grown in different f/2 media. n = 3. C, Cell growth during S1-8 in f/2-NH_4Cl. n = 3. D, Cell dry weight during S1-8 in f/2-NH_4Cl. n = 3.
