# Supplementary material for: Enhancing oil production and harvest by combining the marine alga Nannochloropsis oceanica and the oleaginous fungus Mortierella elongata
Source: Biotechnol Biofuels. 2018 Jun 22;11:174. doi: 10.1186/s13068-018-1172-2 (PMC6013958; doi:10.1186/s13068-018-1172-2)
Supplement: Supplementary file 5 — Additional file 5: Figure S4. Triacylglycerol accumulation during prolonged-incubation in f/2 medium supplemented with or without sodium bicarbonate. [file 13068_2018_1172_MOESM5_ESM.pdf]

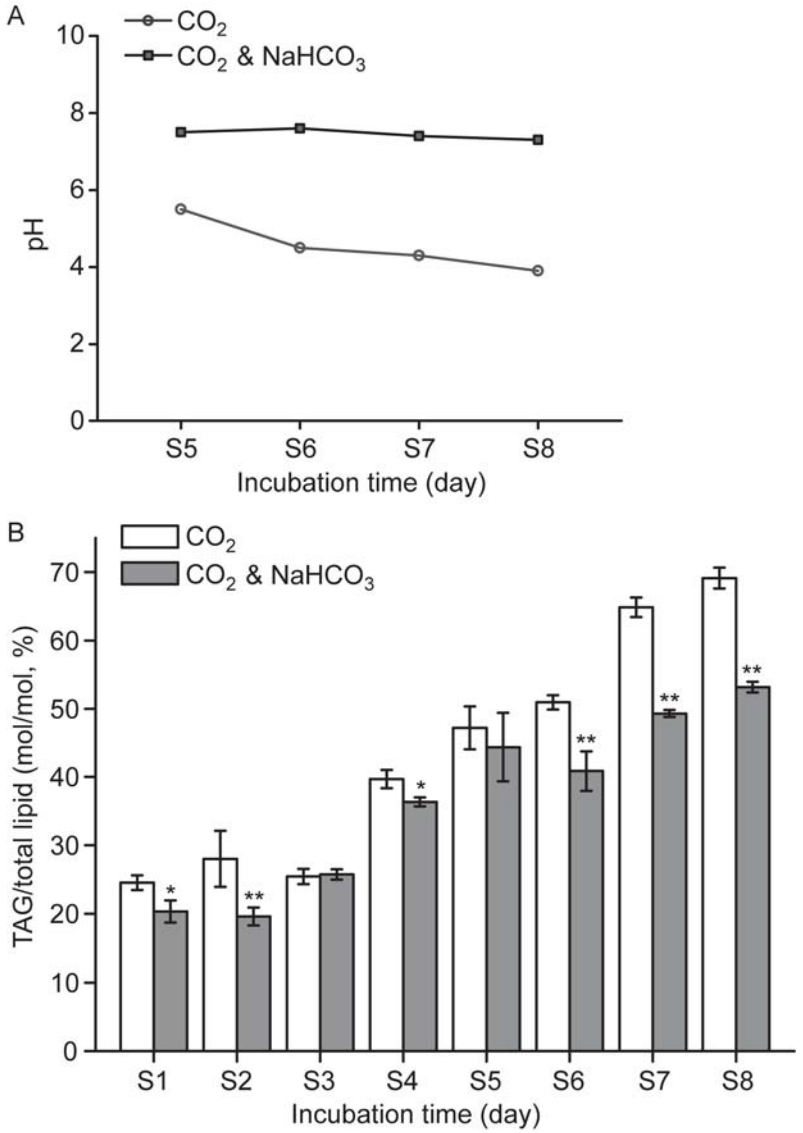

Figure S4. Triacylglycerol accumulation during prolonged-incubation in f/2-NH_4Cl medium supplemented with or without sodium bicarbonate. *N. oceanica* cells were inoculated and incubated in f/2-NH_4Cl medium (with or without $NaHCO_3$) in ePBRs and sparged with air enriched to 5% CO_2 at 0.37 L min^{-1} for 2 min per h. S1 to 8, day 1 to 8 after the cultures reached stationary phase. A, pH measurement from S5 to S8. B, Analysis of TAG content during prolonged incubation. The results are the average of three biological replicates and error bars indicate standard deviation. Asterisks indicate significant difference between CO_2 and CO_2 & $NaHCO_3$. **, $P < 0.01$; *, $P < 0.05$; n=3.
