# Supplementary material for: Enhancing oil production and harvest by combining the marine alga Nannochloropsis oceanica and the oleaginous fungus Mortierella elongata
Source: Biotechnol Biofuels. 2018 Jun 22;11:174. doi: 10.1186/s13068-018-1172-2 (PMC6013958; doi:10.1186/s13068-018-1172-2)
Supplement: Supplementary file 6 — Additional file 6: Figure S5. Maps of the plasmids used for the generation of N. oceanica DGTT5-overexpressing strains. [file 13068_2018_1172_MOESM6_ESM.pdf]

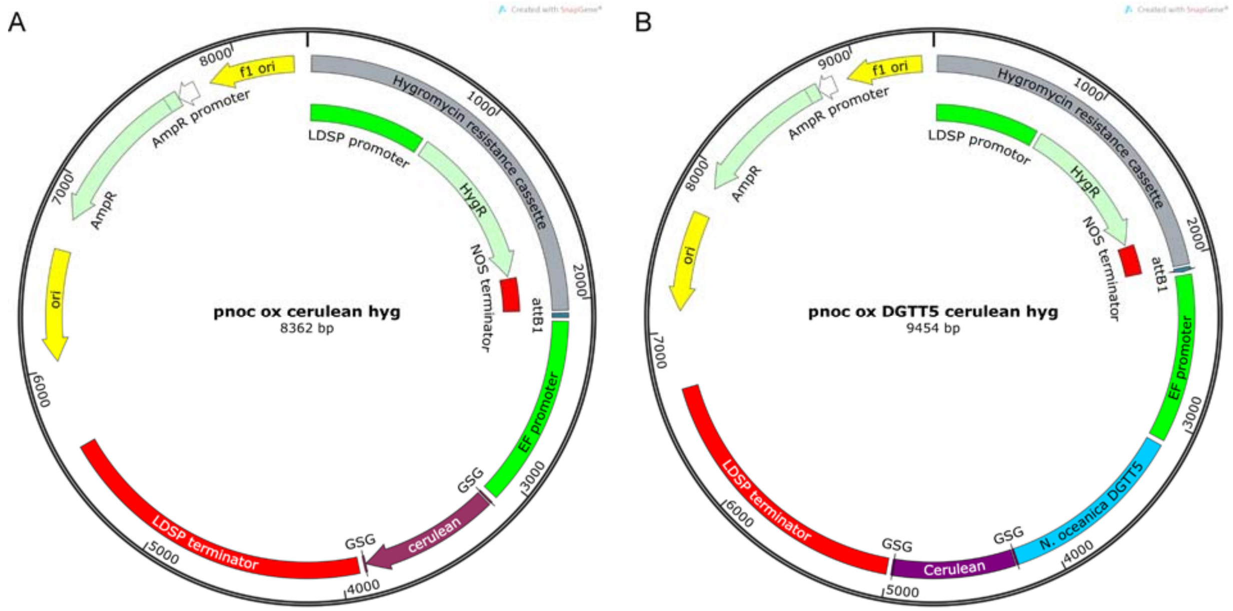

Figure S5. Maps of the plasmids used for the generation of *N. oceanica DGTT5*-overexpressing strains. A, pnoc ox cerulean hyg vector control. B, pnoc ox DGTT5 cerulean hyg for overexpressing *DGTT5*.
