# Supplementary material for: Enhancing oil production and harvest by combining the marine alga Nannochloropsis oceanica and the oleaginous fungus Mortierella elongata
Source: Biotechnol Biofuels. 2018 Jun 22;11:174. doi: 10.1186/s13068-018-1172-2 (PMC6013958; doi:10.1186/s13068-018-1172-2)
Supplement: Supplementary file 7 — Additional file 7: Figure S6. Increasing triacylglycerol content in N. oceanica by the overexpression of N. oceanica DGTT5 encoding acyl-CoA:diacylglycerol acyltransferase DGTT5. [file 13068_2018_1172_MOESM7_ESM.pdf]

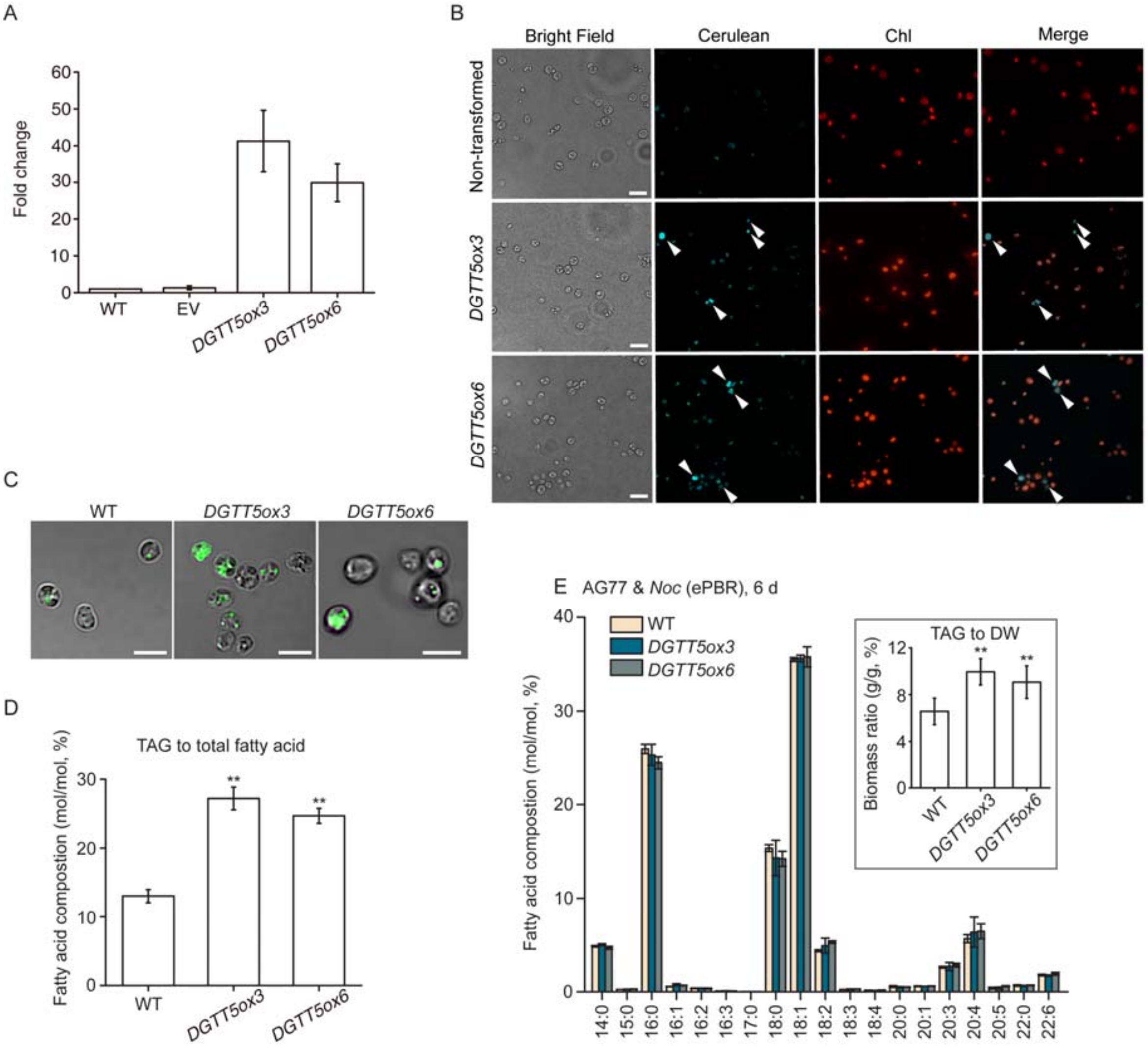

Figure S6. Increasing triacylglycerol content in *N. oceanica* by the overexpression of *N. oceanica DGTT5* encoding acyl-CoA:diacylglycerol acyltransferase DGTT5. *N. oceanica DGTT5*-overexpressing strains *DGTT5ox3* and *DGTT5ox6* were generated and examined by quantitative RT-PCR (qRT-PCR) as previously described (Zienkiewicz et al., 2017). A, Relative expression of *N. oceanica DGTT5* in the wild type (WT), empty vector control (EV), and two *DGTT5*-overexpressing strains *DGTT5ox3* and *DGTT5ox6*, analyzed by the $2^{-\Delta\Delta C_T}$ method using *ACTIN* as the reference gene. The values were normalized to the level of the WT. Each data point represents the average of three biological replicates and error bars means standard deviation (n = 3). B, Detection of Cerulean fluorescent protein that was fused to the *N. oceanica* DGTT5 by confocal

microscopy (emission 420-440 nm). Arrow heads indicate Cerulean fluorescence. Chl, autofluorescence of chloroplasts. C, Detection of lipid droplets by BODIPY staining. The WT and *DGTT5ox* cells were incubated in shaker flasks containing f/2 medium. D, TAG content of the WT and *DGTT5ox* strains grown in shaker flasks containing f/2 medium. **, $P < 0.01$; n = 4. E, Fatty acid analyses of TAG in the alga-fungus aggregates incubated in shaker flasks. Biomass ratio of TAG compared to the total cell dry weight (DW) was calculated and shown here. n = 4.
