# Supplementary material for: Enhancing oil production and harvest by combining the marine alga Nannochloropsis oceanica and the oleaginous fungus Mortierella elongata
Source: Biotechnol Biofuels. 2018 Jun 22;11:174. doi: 10.1186/s13068-018-1172-2 (PMC6013958; doi:10.1186/s13068-018-1172-2)
Supplement: Supplementary file 8 — Additional file 8: Table S2. Predicted genes and proteins involved in fatty acid and glycerolipid synthesis in M. elongata AG77. [file 13068_2018_1172_MOESM8_ESM.pdf]

Table S2. Predicted genes and proteins involved in fatty acid and glycerolipid synthesis in *M. elongata* AG77.

**Fatty acid biosynthesis**				
	**Description**	**Name**	**Transcript ID**	**Protein ID**
**Acetyl-CoA carboxylase components**	acetyl-CoA carboxylase	ACC	134167	133928
	acetyl-CoA carboxylase, subunit beta	ACC	67410	67171
	acetyl-CoA carboxylase, subunit beta	ACC	75685	75446
	acetyl-CoA carboxylase, subunit beta	ACC	75799	75560
	malonyl-CoA decarboxylase	MLYCD	100665	100426
	malonyl-CoA decarboxylase	MLYCD	81573	81334
	acyl carrier protein	ACP	128202	127963
	acyl carrier protein	ACP	139468	139229
**Type I fatty acid synthase**	fatty acid synthase	FAS	1805138	1804883
**putative fatty acid synthase components**	malonyl-CoA:ACP malonyltransferase	FabD	144910	144671
	malonyl-CoA:ACP malonyltransferase	FabD	522882	522643
	3-oxoacyl-ACP synthase, KASI/II	FabB/F	115244	115005
	3-oxoacyl-ACP synthase, KASI/II	FabB/F	1878602	1878347
	3-hydroxydecanoyl-ACP dehydratase	FabA	131674	131435
	putative 3-Ketoacyl-ACP reductase	FabG	1769266	1769011
**Elongases**	acyl-CoA elongase	ELO	132697	132458
	acyl-CoA elongase	ELO	134272	134033
	acyl-CoA elongase	ELO	140756	140517
	acyl-CoA elongase	ELO	141020	140781
	acyl-CoA elongase	ELO	14820	14581
	acyl-CoA elongase	ELO	147783	147544
	acyl-CoA elongase	ELO	148635	148396
	acyl-CoA elongase	ELO	165821	165582
	acyl-CoA elongase	ELO	1880273	1880018
**Desaturases**	fatty acid Δ9-desaturase	FADS9	107360	107121
	fatty acid Δ9-desaturase	FADS9	108744	108505
	fatty acid Δ9-desaturase	FADS9	138135	137896
	fatty acid Δ9-desaturase	FADS9	1816261	1816006
	fatty acid Δ6-desaturase	FADS6	134789	134550
	fatty acid Δ6-desaturase	FADS6	158522	158283
	fatty acid desaturase	FAD	140331	140092
	fatty acid desaturase	FAD	1751385	1751130
	fatty acid desaturase	FAD	15652	15413
	fatty acid Δ12-desaturase	FADS12	17302	17063
	fatty acid Δ5-desaturase	FADS5	87849	87610
	fatty acid Δ15-desaturase	FADS15	152410	152171
**Acyl-CoA thioesterase**	acyl-CoA thioesterase	ACOT	14633	14394
	acyl-CoA thioesterase	ACOT	54405	54166

**and synthetase**	acyl-CoA thioesterase	ACOT	561278	561039
	acyl-CoA thioesterase	ACOT	33252	33013
	acyl-CoA synthetase	ACSL	123145	122906
	acyl-CoA synthetase	ACSL	134960	134721
	acyl-CoA synthetase	ACSL	143367	143128
	acyl-CoA synthetase	ACSL	75546	75307
	acyl-CoA synthetase	ACSL	131674	131435
	acyl-CoA synthetase	ACSL	150818	150579
	acyl-CoA synthetase	ACSL	72538	72299
	acyl-CoA synthetase	ACSL	74248	74009
	acyl-CoA synthetase	ACSL	81012	80773
	acyl-CoA synthetase	ACSL	94221	93982
	acyl-CoA synthetase	ACSL	126107	125868
	acyl-CoA synthetase	ACSL	73494	73255
**Glycerolipid biosynthesis**				
	**Description**	**Name**	**Transcript**	**Protein ID**
	aldehyde dehydrogenase	ALDH	14282	14043
	aldehyde dehydrogenase	ALDH	138532	138293
	aldehyde dehydrogenase	ALDH	138027	137788
	aldehyde dehydrogenase	ALDH	145556	145317
	aldehyde dehydrogenase	ALDH	36004	35765
	aldehyde dehydrogenase	ALDH	34024	33785
	alcohol dehydrogenase	ADH	103662	103423
	alcohol dehydrogenase	ADH	144920	144681
	alcohol dehydrogenase	ADH	157172	156933
	alcohol dehydrogenase	ADH	80690	80451
	alcohol dehydrogenase	ADH	150046	149807
	alcohol dehydrogenase	ADH	36977	36738
	alcohol dehydrogenase	ADH	21055	20816
	alcohol dehydrogenase	ADH	84445	84206
	glycerol kinase	GK	95496	95257
	glycerol-3-phosphate dehydrogenase	GPDH	141744	141505
	glycerol-3-phosphate dehydrogenase	GPDH	133004	132765
	glycerol-3-phosphate dehydrogenase	GPDH	143386	143147
	glycero-3-phosphate acyltransferase	GPAT	132665	132426
	glycero-3-phosphate acyltransferase	GPAT	71699	71460
	glycero-3-phosphate acyltransferase	GPAT	136092	135853
	glycero-3-phosphate acyltransferase	GPAT	426195	425956
	glycero-3-phosphate acyltransferase	GPAT	114545	114306
	glycero-3-phosphate acyltransferase	GPAT	156906	156667
	glycero-3-phosphate acyltransferase	GPAT	142242	142003
	glycero-3-phosphate acyltransferase	GPAT	138636	138397
	1-sn-acyl-glycero-3-phosphate acyltransferase	PlsC	133934	133695

	1-sn-acyl-glycero-3-phosphate acyltransferase	PlsC	15247	15008
	phosphatidic acid phosphatase	PAP	72762	72523
	phosphatidic acid phosphatase	PAP	67757	67518
	phosphatidic acid phosphatase	PAP	118493	118254
	phosphatidic acid phosphatase	PAP	143215	142976
	phosphatidic acid phosphatase	PAP	141373	141134
	Lipin like/phosphatidate phosphatase	LPIN	22296	22057
	Lipin like/phosphatidate phosphatase	LPIN	33916	33677
	diacylglycerol kinase	Dgk	32027	31788
	diacylglycerol kinase	Dgk	143293	143054
	diacylglycerol kinase	Dgk	133967	133728
	diacylglycerol kinase	Dgk	111955	111716
	diacylglycerol kinase	Dgk	133379	133140
	diacylglycerol kinase	Dgk	134894	134655
	diacylglycerol acyltransferase	DGAT	102618	102379
	diacylglycerol acyltransferase	DGAT	14740	14501
TAG synthesis	diacylglycerol acyltransferase	DGAT	135508	135269
	phospholipid diacylglycerol acyltransferase	PDAT	872488	872249
